# BMP-7 Enhances Cell Migration and αvβ3 Integrin Expression via a c-Src-Dependent Pathway in Human Chondrosarcoma Cells

**DOI:** 10.1371/journal.pone.0112636

**Published:** 2014-11-12

**Authors:** Jui-Chieh Chen, Shu-Ting Yang, Chih-Yang Lin, Chin-Jung Hsu, Chun-Hao Tsai, Jen-Liang Su, Chih-Hsin Tang

**Affiliations:** 1 Department of Biochemical Science and Technology, National Chiayi University, Chiayi, Taiwan; 2 Graduate Institute of Basic Medical Science, China Medical University, Taichung, Taiwan; 3 National Institute of Cancer Research, National Health Research Institutes, Miaoli County, Taiwan; 4 School of Pharmacy, China Medical University, Taichung, Taiwan; 5 School of Chinese Medicine, College of Chinese Medicine, China Medical University, Taichung, Taiwan; 6 Department of Orthopedic Surgery, China Medical University Hospital, Taichung, Taiwan; 7 Department of Medicine and Graduate Institute of Clinical Medical Science, China Medical University, Taichung, Taiwan; 8 Department of Biotechnology, College of Health Science, Asia University, Taichung, Taiwan; 9 Center for Molecular Medicine, China Medical University Hospital, Taichung, Taiwan; 10 Graduate Institute of Cancer Biology, China Medical University, Taichung, Taiwan; 11 Department of Pharmacology, School of Medicine, China Medical University, Taichung, Taiwan; Instituto Gulbenkian de Ciência, Portugal

## Abstract

Bone morphogenic protein (BMP)-7 is a member of the transforming growth factor (TGF)-beta superfamily, which is originally identified based on its ability to induce cartilage and bone formation. In recent years, BMP-7 is also defined as a potent promoter of cell motility, invasion, and metastasis. However, there is little knowledge of the role of BMP-7 and its cellular function in chondrosarcoma cells. In the present study, we investigated the biological impact of BMP-7 on cell motility using transwell assay. In addition, the intracellular signaling pathways were also investigated by pharmacological and genetic approaches. Our results demonstrated that treatment with exogenous BMP-7 markedly increased cell migration by activating c-Src/PI3K/Akt/IKK/NF-κB signaling pathway, resulting in the transactivation of αvβ3 integrin expression. Indeed, abrogation of signaling activation, by chemical inhibition or expression of a kinase dead form of the protein attenuated BMP-7-induced expression of integrin αvβ3 and cell migration. These findings may provide a useful tool for diagnostic/prognostic purposes and even therapeutically in late-stage chondrosarcoma as an anti-metastatic agent.

## Introduction

Chondrosarcoma, the second most common type of bone cancer, is a heterogeneous group of neoplasms that are characterized by the production of cartilage matrix. High-grade chondrosarcoma is more aggressive and is more likely to metastasize to other parts of the body, leading to poor prognosis and lethality. To date, surgical resection remains the only effective therapy for chondrosarcoma, since conventional chemotherapy and radiotherapy are largely ineffective [1], [2]. It is therefore urgent need to develop more effective treatments against chondrosarcoma.

Bone morphogenic proteins (BMPs) belong to the transforming growth factor-β (TGF-β) superfamily, which discovered because of their remarkable ability to induce endochondral bone formation [3]. BMPs exert their biological function by binding to type I and type II serine-threonine kinase receptors, and transduce signals through both Smad-dependent and -independent pathways [4], [5], [6]. Aberrations in BMPs signaling have also been identified in various neoplasms, which are involved in tumor aggressiveness [7], [8]. In conventional central chondrosarcoma, BMP signaling pathway is active and that the activity correlates to the histopathological grade [9]. To date, over 20 members of the BMP subgroup have been identified [4].

Among the BMPs, BMP-7 (previously called osteogenic protein-1, OP-1) is one of the best characterized osteogenic factors, which has been reported to induce cartilage and bone formation in animal models and enhance bone repair in clinical studies [10], [11], [12]. A previous study has shown that BMP-7 is significantly higher expressed in chondrosarcoma, while it is not detected or found at very low expression levels in normal cartilage samples [9]. In addition, another study also found that BMP-7 levels are higher in the high-grade chondrosarcoma than in the low-grade one [13]. However, the function of BMP-7 on chondrosarcoma cells has not yet been investigated.

Accumulating evidence reveals that high-level expression of BMP-7 correlates with increased invasion and metastasis in various malignancies, including breast cancer [14], colorectal cancer [15], prostate cancer [16], esophageal cancer [17], gastric cancer [3], lung cancer [18], liver cancer [6], and melanoma [5]. Although the roles of BMP-7 have emerged as an important factor in the regulation of cell motility across diverse cancer, the influence of BMP-7 on the motility of chondrosarcoma cells still remains largely unknown. In the present study, we explored the molecular mechanism by which BMP-7 signaling to regulate cell motility in human chondrosarcoma cells. Additionally, previous studies have shown that multiple non-Smad pathwayss, including c-Src, phosphoinositide 3-kinase (PI3K)/Akt, or nuclear factor (NF)-κB were turned on by BMP [19], [20].

Integrins is one of the most important factors, which play critical roles in cancer cell migration, invasion, and metastasis contributing to tumor progression. Activation and elevated expression of integrin have been implicated in the induction of cell migration in a wide variety of human cancers [21], [22]. It should be noted that the expression of integrin was previously shown to be modulated by the activation of PI3K, Akt, and NF-κB [23], [24]. We have thus determined the role of the c-Src, PI3K/Akt, and NF-κB pathways in BMP-7-induced cellular motility in chondrosarcoma cells, especially at the level of integrin expression.

## Materials and Methods

### Materials

Recombinant human BMP-7 was purchased from PeproTech (Rocky Hill, NJ). Mouse monoclonal antibody specific for αvβ3 integrin were purchased from Millipore (Bedford, MA). Fluorescein isothiocyanate (FITC)-conjugated goat anti-mouse secondary antibody was purchased from Leinco Technology Inc. (St Louis, MO). Rabbit polyclonal antibodies specific for c-Src, p85, p-Akt (Ser^473^), Akt, p-IKKα/β (Ser^180/181^), IKK, and p65, as well as horseradish peroxidase-conjugated anti-mouse and anti-rabbit IgG, were purchased from Santa Cruz Biotechnology (Santa Cruz, CA). Rabbit polyclonal antibody specific for p-c-Src (Tyr^416^), p-p85 (Tyr^458^), and p-p65 (Ser^536^) were purchased from Cell Signaling and Neuroscience (Danvers, MA). PP2, Akt inhibitor, LY294002, wortmannin, pyrrolidine dithiocarbamate (PDTC) and N-tosyl-L phenylalanyl-chloromethyl ketone (TPCK) were purchased from Calbiochem (San Diego, CA). All inhibitors were used at a final concentration of 10 µM. The c-Src dominant negative mutant was a gift from Dr. S. Parsons (University of Virginia Health System, Charlottesville, VA). The p85 (Δp85; deletion of 35 amino acids from residues 479 to 513 of p85) and Akt (Akt K179A) dominant-negative mutants were gifts from Dr. W.M. Fu (National Taiwan University, Taipei, Taiwan). The IKKα (KM) and IKKβ (KM) mutants were provided by Dr. H. Nakano (Juntendo University, Tokyo, Japan). The NF-κB luciferase plasmid was purchased from Stratagene (La Jolla, CA, USA). pSV-β-galactosidase vector and luciferase assay kit were purchased from Promega (Madison, MA, USA). Lipofectamine 2000 transfection reagent was purchased from Invitrogen (Carlsbad, CA, USA). All other reagents were obtained from Sigma-Aldrich (St. Louis, MO).

### Cell culture

The human chondrosarcoma cell line (JJ012) was kindly provided by the laboratory of Dr. Sean P. Scully (University of Miami School of Medicine, Miami, FL) and originated from Dr. Joel Block (Rush University Medical Center, Chicago, Illinois) [25], [26]. JJ012 cells were cultured in a complete medium containing Dulbecco's modified Eagle's medium (DMEM)/α-minimum essential medium (α-MEM) supplemented with 10% fetal bovine serum and 100 units/ml penicillin/streptomycin at 37°C in a humidified chamber in 5% CO_2_.

### Migration assay

The migration assay was performed using Transwell (Costar, NY; pore size, 8 mm). Approximately 1.5×10^4^ cells in 200 µl of serum-free medium were placed in the upper chamber, and 300 µl of the same medium was placed in the lower chamber. The plates were incubated for 24 h at 37°C in 5% CO_2_, cells were then fixed in 3.7% formaldehyde for 15 min and stained with 0.05% crystal violet in PBS for 15 min. Cells on the upper side of the filters were removed with cotton-tipped swabs, and the filters were washed with PBS. Cells on the underside of the filters were examined and counted under a microscope. Each clone was plated in triplicate for each experiment, and each experiment was repeated at least three times.

### Quantitative real-time PCR

Total RNA was extracted from chondrosarcoma cells using a TRIzol kit (MDBio Inc., Taipei, Taiwan). The reverse transcription reaction was performed using 2 µg of total RNA that was reverse transcribed into cDNA using an oligo(dT) primer. Quantitative real-time polymerase chain reaction (qPCR) analysis was carried out using TaqMan one-step PCR Master Mix (Applied Biosystems, Foster City, CA, USA). Total complementary DNA (100 ng/25µl reaction) was mixed with sequence-specific primers and TaqMan probes according to the manufacturer's instructions. Sequences for all target gene primers and probes were purchased commercially (GAPDH was used as internal control) (Applied Biosystems). The q-PCR assays were carried out in triplicate using a StepOnePlus sequence detection system. The cycling conditions were 10 min of polymerase activation at 95°C, followed by 40 cycles at 95°C for 15 s and 60°C for 60 s.

### Flow cytometry analysis

Human chondrosarcoma cells were plated in six-well dishes. The cells were then washed with PBS and detached with trypsin at 37°C. Cells were fixed for 10 min in PBS containing 1% paraformaldehyde. After rinsing in PBS, the cells were incubated with mouse anti-human antibody against αvβ3 integrin (1∶100) for 1 h at 4°C. Cells were then washed again and incubated with fluorescein isothiocyanate-conjugated goat anti-mouse secondary IgG (1∶150; Leinco Tec. Inc., St. Louis, MO, USA) for 45 min and analyzed by flow cytometry using FACS Calibur and CellQuest software (BD Biosciences, Palo Alto, CA, USA).

### Western blot analysis

Proteins in the total cell lysate (40 µg of protein) were separated on a 10% sodium dodecyl sulfate–polyacrylamide gel electrophoresis gel (SDS-PAGE) and electrotransferred onto a polyvinylidene difluoride (PVDF) membranes. The blots were blocked with 5% skim milk for 1 h at room temperature and then probed with the indicated primary antibodies for 1 h at room temperature. After three washes by 0.1% Tween 20 and Tris buffer saline Tween20 (TBST), the blots were incubated with the appropriate secondary antibodies conjugated to horseradish peroxidise for 1 h at room temperature. The blots were visualized by ECL reagents (PerkinElmer, MA, USA) and autoradiography.

### Transfection

Transient transfection of dominant-negative mutants (0.5 µg) was carried out using Lipofectamine 2000, according to the manufacturer's instructions.

### Luciferase reporter assay

Human chondrosarcoma cells were transfected with NF-κB luciferase plasmid using Lipofectamine 2000. At 24 h after transfection, the cells were pretreated with inhibitors for 30 min, and then, BMP-7 or vehicle was added for 24 h. Cell extracts were then prepared, and luciferase and β-galactosidase activities were measured.

### Electrophoretic mobility shift assay

Electrophoretic mobility shift assay was performed by using EMSA “gel shift” kit (Panomics, Redwood City, CA) according to the manufacturer's protocol. NF-κB consensus oligonucleotide probe (5′-AGTTGAGGGGACTTTCCCAGGC-3′) was used. Nuclear extract (3 µg) of cells was incubated with poly d(I–C) at room temperature for 5 min. The nuclear extract was then incubated with biotin-labeled probes and the incubated at room temperature for 30 min. After electrophoresis on a 6% polyacrylamide gel, the samples on gel were transferred onto a presoaked Immobilon-Ny + membrane (Millipore, Billerica, MA). The membrane was baked at 80°C for 1 h, cross-linked in an oven for 3 min and then developed by adding the blocking buffer and streptavidin–horseradish peroxidase conjugate.

### Statistics

Data are presented as mean ± standard error of the mean (SEM). Statistical comparison of two groups was performed using the Student's *t* test. Statistical comparisons of more than two groups were performed using one-way analysis of variance (ANOVA) with Bonferroni's *post-hoc* test. In all cases, *p*<0.05 was considered significant.

## Results

### BMP-7 induces the migration activity of human chondrosarcoma cells via up-regulation of integrin αvβ3 expression

**Figure 1:**
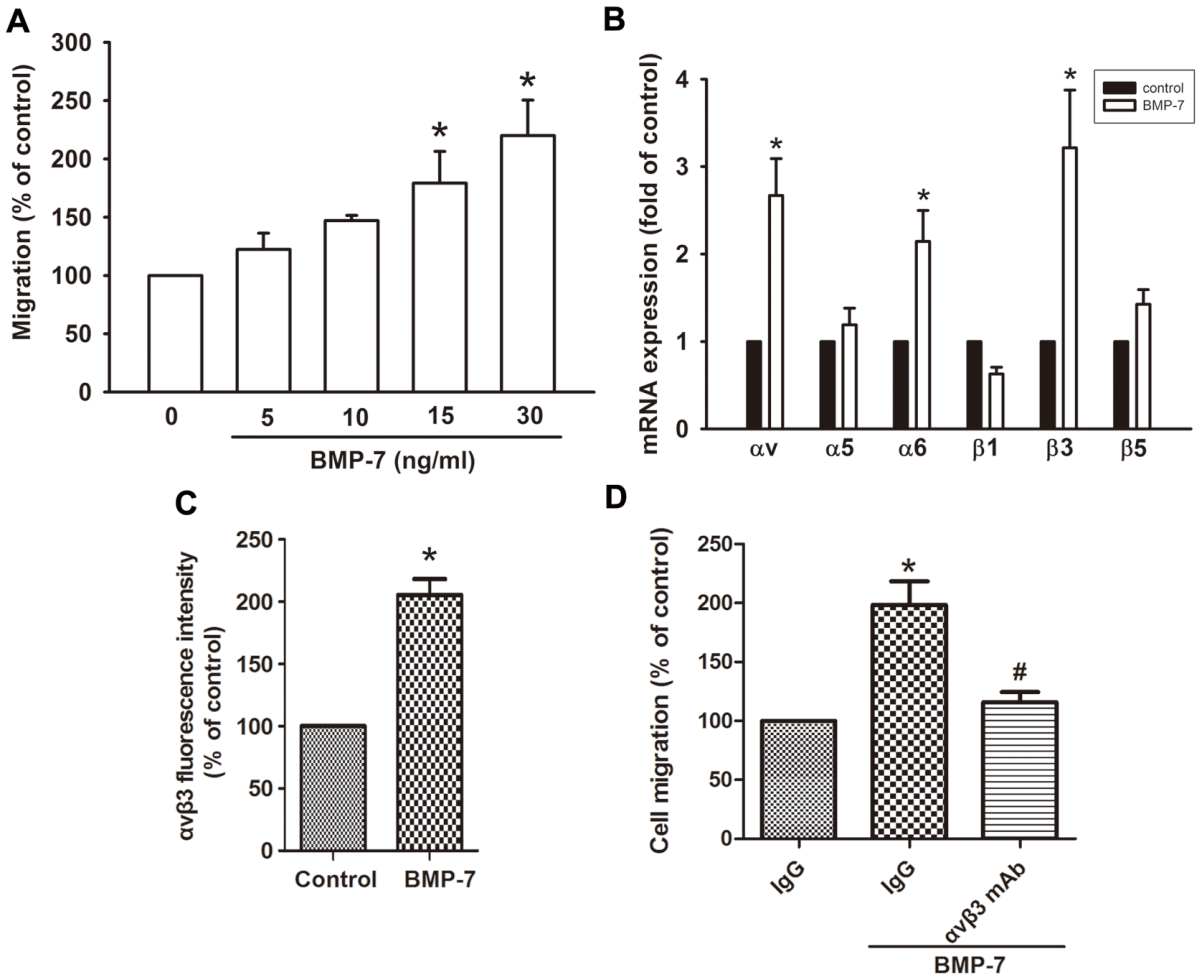
BMP-7 enhanced cell migration through up-regulation of integrin αvβ3 expression. (A) JJ012 cells were incubated with BMP-7 (5–30 ng/ml) for 24 h, and *in vitro* migration was measured with the Transwell after 24 h (n = 6). (B) JJ012 cells were incubated with BMP-7 (30 ng/ml) for 24 h, and mRNA expression of αv, α5, α6, β1, β3, and β5 integrin were examined by q-PCR (n = 6). (C) Cells were incubated with or without BMP-7 for 24 h and the protein expression levels of integrin αvβ3 were examined by flow cytometry analysis (n = 5). (D) Cells were pretreated with αvβ3 monoclonal antibody (10 µg/ml) for 30 min followed by stimulation with BMP-7. The *in vitro* migration activity measured after 24 h (n = 5). Results are expressed as the mean ± SEM. *p<0.05, compared to basal expression levels.

We initially assessed the effects of BMP-7 on the migration activity in human chondrosarcoma cells. The treatment of JJ012 cells with BMP-7 resulted in a dose-dependent increase in cell migration, as assessed by Transwell assay (Fig. 1A). Accumulating evidence reveals that increased integrin expression and signaling are implicated in cancer cell migration, invasion, and metastasis in human chondrosarcoma cells [1]. We therefore hypothesized that BMP-7 may promote cell migration by increasing the expression of specific integrins. The q-PCR analysis showed that BMP-7 induced αv, α6, and β3 but not α5, β1, and β5 integrin expression (Fig. 1B). However, human β3 pairs only with human αv and does not assemble a heterodimer with α6 [22], [27]. The expression of integrin αvβ3 was also validated by flow cytometry analysis (Fig. 1C). To further confirm the effect of BMP-7 on migration through αvβ3 integrin, JJ012 cells pre-treated with anti-αvβ3 monoclonal antibody markedly inhibited the BMP-7-induced cell migration (Fig. 1D). These results suggest that BMP-7 increased cell migration through up-regulation of integrin αvβ3 in chondrosarcoma cells.

### The c-Src is involved in the BMP-7-mediated increase of integrin αvβ3 expression and migration ability

**Figure 2:**
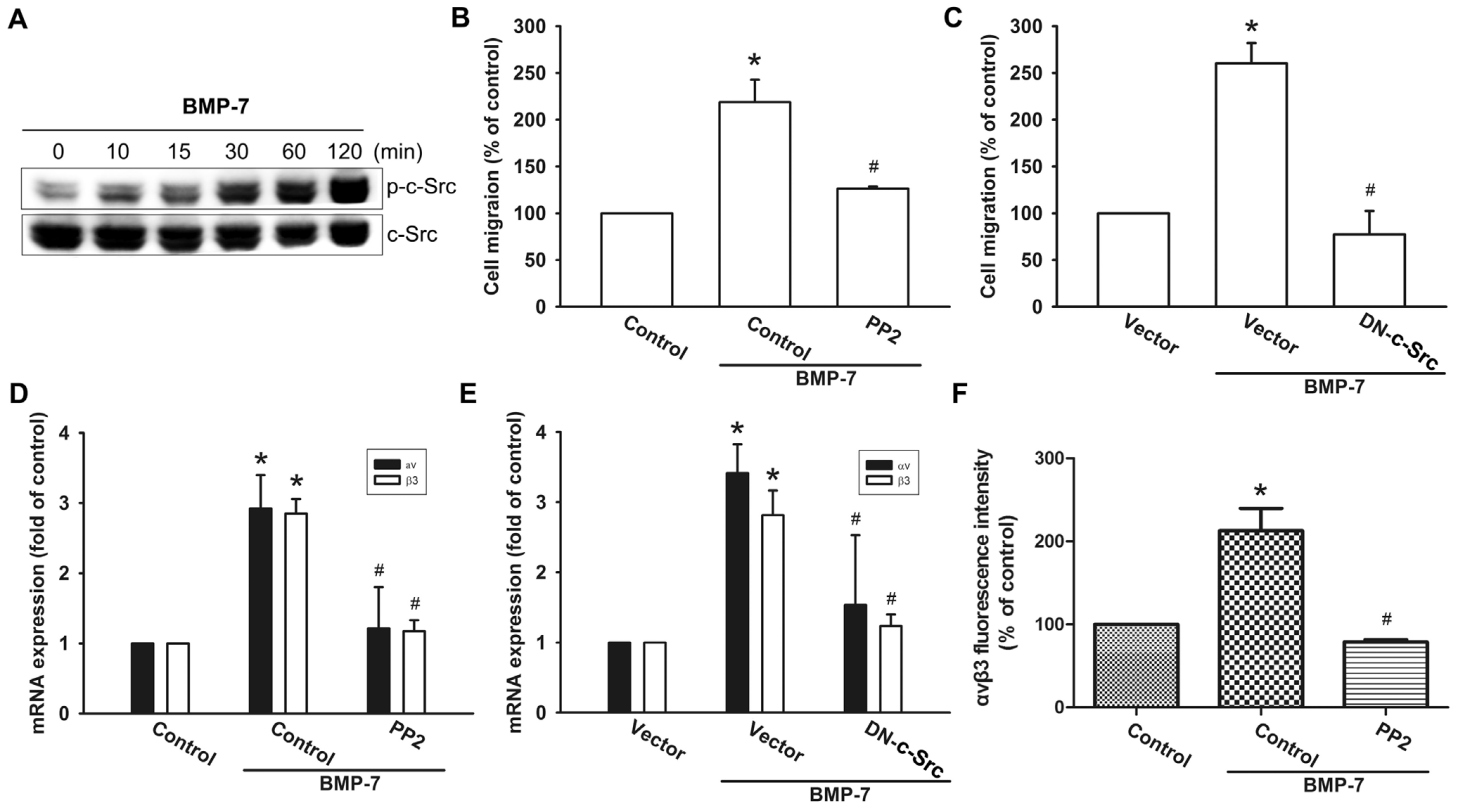
The c-Src is required for in BMP-7-induced cell migration and integrin αvβ3 expression in human chondrosarcoma cells. (A) JJ012 cells were incubated with BMP-7 for the indicated time intervals and the phosphorylation of the c-Src were determined by western blot. Data are representative of at least three independent experiments. (B–E) Cells were pretreated with PP2 (10 µM) for 30 min or co-transfected with c-Src mutant for 24 h followed by stimulation with BMP-7 for 24 h, and *in vitro* migration and integrin αvβ3 expression was measured by Transwell (n = 4) and qPCR (n = 4). (F) Cells were pretreated with PP2 for 30 min and then incubated with BMP-7 for 24 h. The protein levels of integrin αvβ3 were determined by flow cytometry analysis (n = 5). Results are expressed as the mean ± SEM. *p<0.05, compared to basal expression levels. #*p*<0.05, compared to expression levels in the BMP-7-treated group.

It has been demonstrated that c-Src mediated cell motility in cancer cells [28], [29], [30]. We sought to investigate whether the c-Src is involved in BMP-7-mediated up-regulation of integrin αvβ3 expression. As shown in Fig. 2A, treatment of JJ012 cells with BMP-7 induced a significant increase in phosphorylation of c-Src in a time-dependent fashion. To determine whether c-Src is involved in BMP-7-mediated cell migration and integrin αvβ3 expression, JJ012 cells were pre-treated with the c-Src inhibitor or transfected with c-Src mutant before starting treatment with BMP-7. The results revealed that BMP-7 was not able to promote cell migration when c-Src was inhibited by PP2 (Fig. 2B) and c-Src mutant (Fig. 2C). In addition, abrogation of c-Src activation, by chemical inhibition or expression of a kinase dead form of the protein also attenuated BMP-7-induced expression of integrin αvβ3 at mRNA levels, as determined by q-PCR analysis (Fig. 2D, E). BMP-7-induced expression of integrin αvβ3, at protein levels, was significantly decreased in the presence of c-Src inhibitors, as assessed by flow cytometry analysis (Fig. 2F). Taken together, these data suggest that c-Src activation may be involved in BMP-7-induced expression of integrin αvβ3 to enhance migration in human chondrosarcoma cells.

### Inhibiting PI3K/Akt activity blocks BMP-7-induced integrin αvβ3 expression and migration

**Figure 3:**
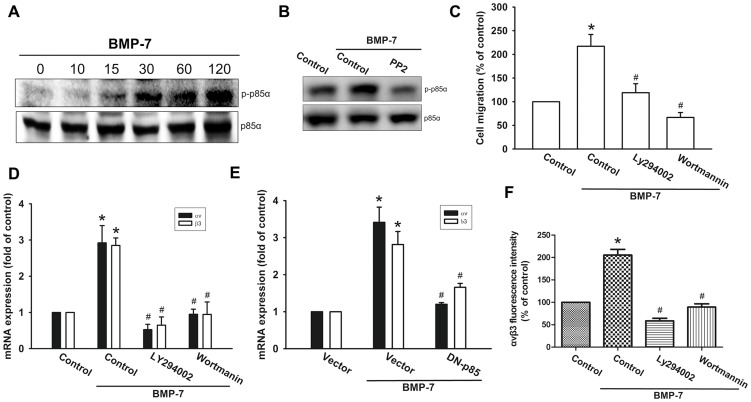
PI3K (p85α) is involved in BMP-7-induced migration and integrin αvβ3 expression. (A–B) JJ012 cells were incubated with BMP-7 for indicated time intervals or pretreated with PP2 for 30 min followed by treatment with BMP-7 for 2 h. The levels of p-p85α and p85α were measured by Western blot. Data are representative of at least three independent experiments. (C) Cells were pretreated with Ly294002 (10 µM) or wortmannin (10 µM) followed by stimulation with BMP-7 for 24 h, and *in vitro* migration was measured by Transwell (n = 4). (D–F) Cells were pretreated with PI3K inhibitor Ly294002 (10 µM) or wortmannin (10 µM) for 30 min or co-transfected with p85 mutant for 24 h followed by incubation with BMP-7 for 24 h. The expression of integrin αvβ3 was measured by q-PCR (n = 4) and flow cytometry (n = 5). Results are expressed as the mean ± SEM. *p<0.05, compared to basal expression levels. #*p*<0.05, compared to expression levels in the BMP-7-treated group.

**Figure 4:**
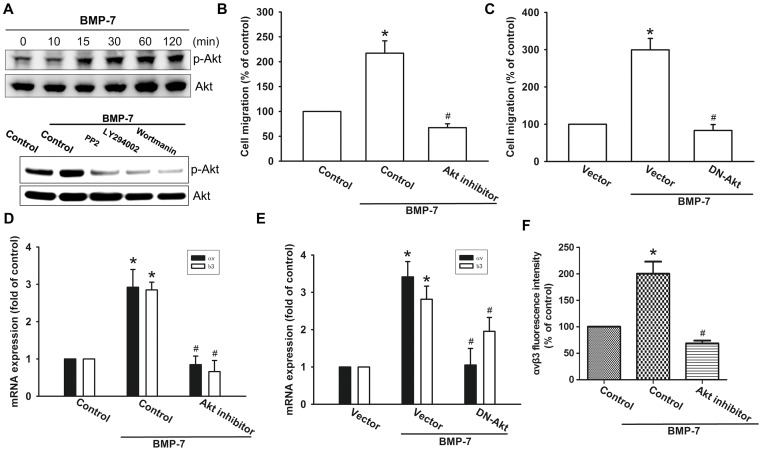
Akt is involved in BMP-7-mediated migration and up-regulation of integrin αvβ3 in human chondrosarcoma cells. (A) JJ012 cells were incubated with BMP-7 for indicated time intervals (upper panel) or pretreated with PP2, Ly294002, or wortmannin for 30 min followed by treatment with BMP-7 for 2 h (lower panel). The levels of p-Akt and Akt were measured by Western blot. Data are representative of at least three independent experiments. (B–E) Cells were pretreated with Akt inhibitor (10 µM) for 30 min or co-transfected with Akt mutant for 24 h, and then incubated with BMP-7 for 24 h. The *in vitro* migration and integrin αvβ3 expression was measured by Transwell (n = 4) and q-PCR (n = 4). (F) The effect of Akt inhibitor on BMP-7-induced up-regulation of integrin αvβ3 at protein level was determined by flow cytometry analysis (n = 5). Results are expressed as the mean ± SEM. *p<0.05, compared to basal expression levels. #*p*<0.05, compared to expression levels in the BMP-7-treated group.

Previous studies have indicated that PI3K/Akt pathway was activated in response to BMP-7 [31], [32]. Therefore, we sought to determine whether the PI3K-Akt pathway is activated and involved in BMP-7-mediated increase of integrin αvβ3 expression and cell migration in human chondrosarcoma cells. As shown in Fig. 3A, treatment of JJ012 cells with BMP-7 resulted in time dependent phosphorylation of p85. However, pretreatment of cells with c-Src inhibitor reduced phosphorylated p85 levels, suggesting that p85 is the downstream target of c-Src (Fig. 3B). Furthermore, pretreatment of cells with the PI3K inhibitor (Ly294002 or wortmannin) abrogated BMP-7-induced in an increase in cell migration (Fig. 3C) and αvβ3 integrin mRNA expression (Fig. 3D). Similar results were also obtained by transfection of cells with p85 mutant, which inhibited BMP-7-mediated αvβ3 integrin mRNA expression (Fig. 3E). Further, PI3K inhibitor (Ly294002 or wortmannin) also significantly inhibited BMP-7-induced the protein levels of integrin αvβ3 expression, as assessed by flow cytometry analysis (Fig. 3F). Next, to examine the role of Akt activation in cancer migration and integrin up-regulation, we inspected Akt phosphorylation in response to BMP-7 treatment. As shown in the upper panel of Fig. 4A, stimulation of JJ012 cells with BMP-7 led to a time-dependent increase in Akt phosphorylation. Inhibition of upstream kinase by PP2, Ly294002, or wortmannin suppressed BMP-7-induced Akt activation (Fig. 4A, lower panel). Furthermore, inhibition of Akt activation, by chemical inhibitors or transfection of cells with a kinase dead form of Akt attenuated BMP-7-induced cell migration (Fig. 4B, C) and expression of integrin αvβ3 at mRNA levels (Fig. 4D, E). Moreover, Akt inhibitor also significantly inhibited BMP-7-induced the protein levels of integrin αvβ3 expression (Fig. 4F). These data suggest that PI3K/Akt activity is involved in BMP-7-induced integrin αvβ3 expression to promote cell migration.

### NF-κB signaling pathway is involved in the BMP-7-mediated integrin upregulation and migration activity

**Figure 5:**
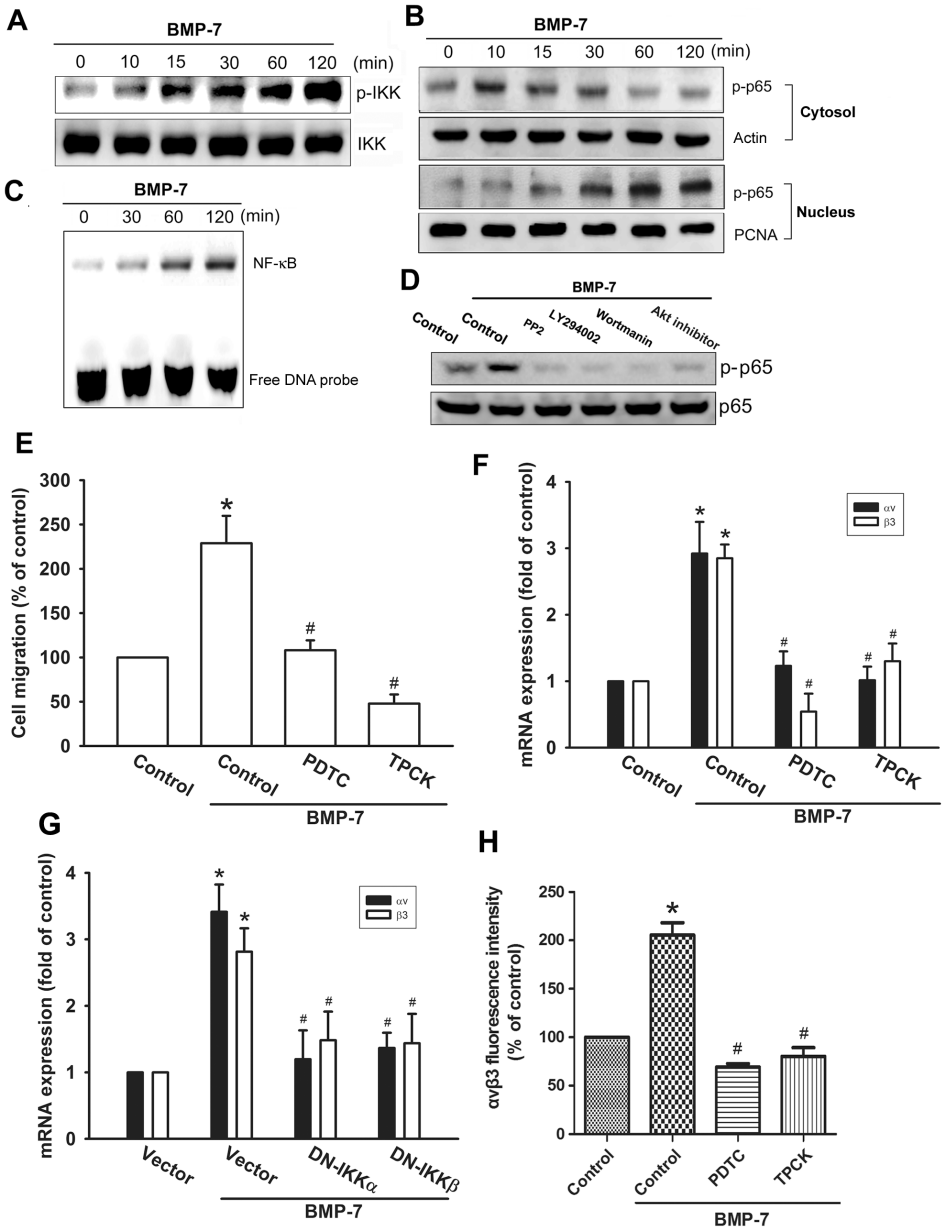
The IKK/NF-κB activation pathway is required for BMP-7-induced cell migration and up-regulation of integrin αvβ3. (A&B) JJ012 cells were incubated with BMP-7 for indicated time intervals. The phosphorylation status and total levels of IKK were measured by Western blot (A). The phosphorylated p65 in cytosol and nucleus were measured by Western blot. The actin and proliferating cell nuclear antigen (PCNA) was used as a loading control for cytosol and nuclear extract, respectively (B). (C) Cells were incubated with BMP-7 for indicated time intervals, and electrophoretic mobility shift assay was performed as described in the Materials and Methods Section. (D) Cells pretreated with PP2, Ly294002, wortmannin, or Akt inhibitor for 30 min followed by treatment with BMP-7 for 2 h. The levels of p-p65 and p65 were measured by Western blot. Data are representative of at least three independent experiments. (E) Cells were pretreated with PDTC (10 µM) or TPCK (10 µM) followed by stimulation with BMP-7 for 24 h, and *in vitro* migration was measured by Transwell (n = 4). (F–H) Cells were pretreated with PDTC (10 µM) or TPCK (10 µM) for 30 min or co-transfected with IKKα and IKKβ mutant for 24 h followed by incubation with BMP-7 for 24 h. The expression of integrin αvβ3 was measured by q-PCR (F, G) (n = 4) and flow cytometry (H) (n = 5). Results are expressed as the mean ± SEM. *p<0.05, compared to basal expression levels. #*p*<0.05, compared to expression levels in the BMP-7-treated group.

**Figure 6:**
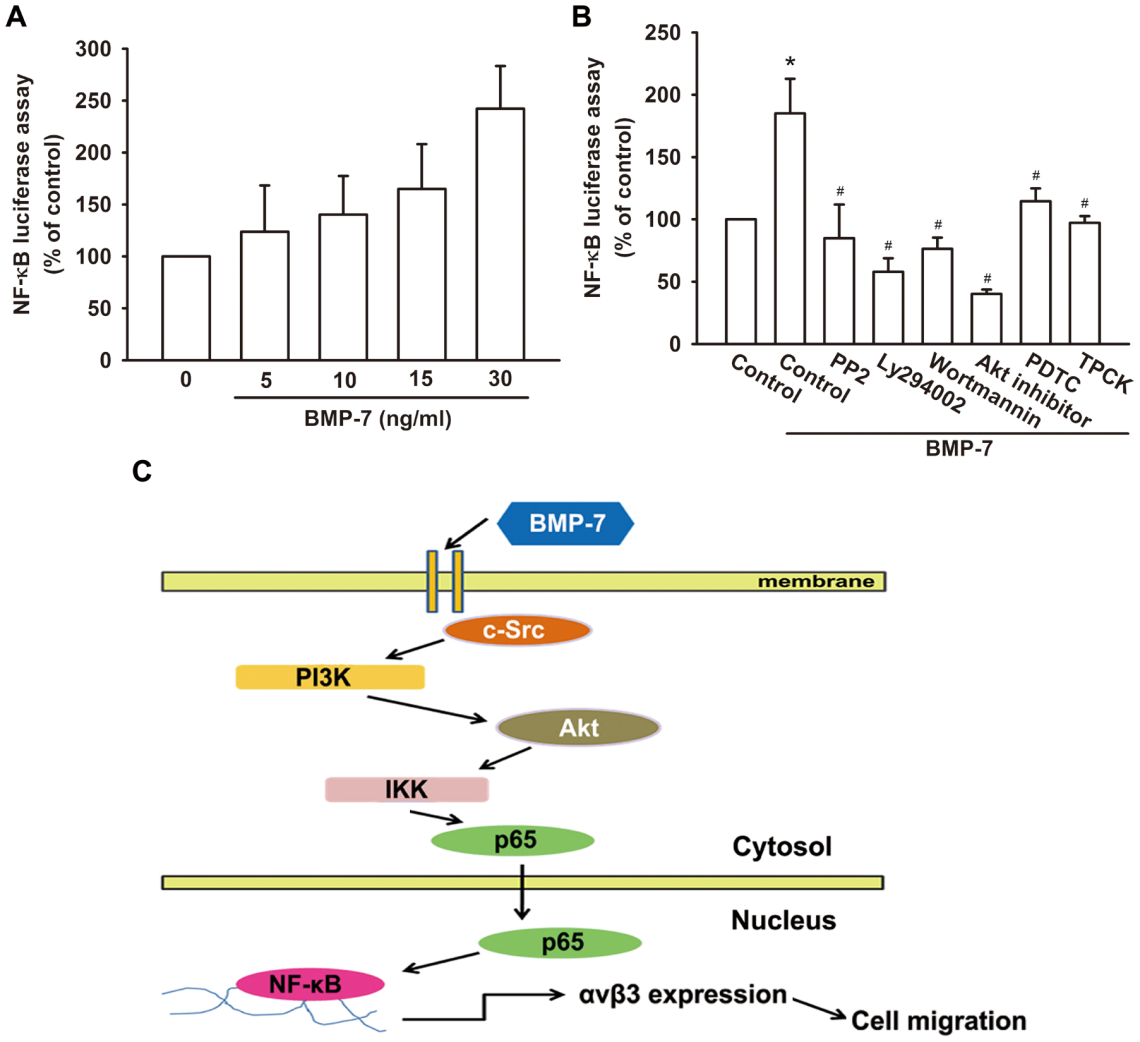
BMP-7 induced activation of NF-κB through c-Src/PI3K/Akt pathway. (A) JJ012 cells were transfected with NF-κB-luciferase reporter for 24 h and then treated with BMP-7 in a dose-dependent manner for 24 h (n = 5). (B) The transfected JJ012 cells pretreated with PP2, Ly294002, wortmannin, Akt inhibitor, PDTC, or TPCK, followed by stimulation with BMP-7 for 24 h. Equal amounts of cell extract were assayed for dual-luciferase activity (n = 5). (C) Proposed scheme for BMP-7-stimulated signaling involved in up-regulation of integrin αvβ3 expression, leading to enhanced cell migration. Results are expressed as the mean ± SEM. *p<0.05, compared to basal expression levels. #*p*<0.05, compared to expression levels in the BMP-7-treated group.

Previously studies have reported that NF-κB is able to regulate many of the genes differentially expressed and implicated in cell migration and invasion [33]. We then investigated whether the activation of NF-κB is critical for BMP-7-mediated increase of integrin αvβ3 expression and cell migration. The results revealed that BMP-7 is able to stimulate a time-dependent phosphorylation of IKK (Fig. 5A). Stimulation of cells with BMP-7 promoted p65 phosphorylation in cytosol at 10 min and translocation into nucleus at 15–120 min (Fig. 5B). In addition, BMP-7 also increased NF-κB-specific DNA–protein complex formation by analyzing electrophoretic mobility shift assay (Fig. 5C). In contrast, BMP-7-induced activation of p65 was markedly suppressed by pre-treatment with upstream pathway inhibitor, including PP2, Ly294002, wortmannin, and Akt inhibitor, implying that NF-κB is the downstream target of c-Src/PI3K/Akt (Fig. 5D). To examine whether NF-κB activation is involved in BMP-7-induced cell migration, the NF-κB inhibitors, PDTC and TPCK, were used. As shown in Fig. 5E, cells pretreated with the NF-κB inhibitors attenuated BMP-7-induced migration capability of chondrosarcoma cells. In addition, pre-treatment of cells with PDTC or TPCK also antagonized BMP-7-induced the expression of αvβ3 integrins, at both mRNA (Fig. 5F) and protein levels (Fig. 5G). Similar results were also obtained by transfection of cells with IKKα or IKKβ mutant, which inhibited BMP-7-induced αvβ3 integrin mRNA expression (Fig. 5H). To further evaluate the c-Src/PI3K/Akt signaling pathway involved in BMP-7-induced NF-κB activation, JJ012 cells were transiently transfected with NF-κB promoter-luciferase construct as an indicator of NF-κB activation. As shown in Fig. 6A, treatment of cells with BMP-7 caused an increase in NF-κB-luciferase activity in a dose-dependent fashion. However, pretreatment of cells with PP2, Ly294002, wortmannin, Akt inhibitor, PDTC, or TPCK antagonized BMP-7-induced NF-κB-luciferase activity (Fig. 6B). Taken together, these data suggest that activation of c-Src, PI3K, Akt, and IKK are required for BMP-7-induced NF-κB activation in human chondrosarcoma cells.

## Discussion

Chondrosarcoma is the second most common malignant bone tumor, which is characterized by the production of cartilage matrix, and their metastatic potential correlates with the histologic grade of the tumor. Furthermore, BMP-7 expression levels are higher in the high-grade sample than in the low-grade one. Like chondrosarcoma, osteosarcoma is an aggressive type of cancer that starts in the bones. Previous data have shown that BMP-7 was highly expressed in human osteosarcoma cell lines but was not expressed in normal osteoblast samples [13]. Another study also found that overexpression of the BMP receptors was related to poor prognosis and metastasis in osteosarcomas, suggesting that BMP pathway may participate in tumor aggressiveness or progression [34]. However, BMP-7 regulation in metastatic behavior and detailed mechanisms are still unclear in chondrosarcoma. In this study, we characterized the effect of BMP-7 on the expression of αvβ3 integrin in human chondrosarcoma cells (JJ012), which may mediate and promote the cell motility. In addition, we also showed that potentiation of αvβ3 integrin by BMP-7 requires the activation of the c-Src, PI3K/Akt, IKK and NF-κB signaling pathway and promotes the expression of αvβ3 integrin and cell migration (Fig. 6C). Another chondrosarcoma cell line, SW1353 cells, was also observed that BMP-7 is able to induce αvβ3 integrin expression and enhance cell migration via the same pathway (Fig. S1). These suggest that c-Src/PI3K/Akt/IKK/NF-κB is a common pathway responsible for αvβ3 integrin expression and cell migration in chondrosarcoma cells.

Distant metastasis is a critical cause for the poor prognosis. Of the factors related to metastasis, BMP-7 has been shown to regulate the aggressiveness of cancer cells. Almost all patients with advanced breast or prostate cancer always develop bone metastases [35]. Previously studies have shown that BMP-7 overexpression is indeed a prognostic indicator for accelerated metastasis formation in breast [36] and prostate cancer [16]. *In vitro* studies showed that treatment with exogenous BMP-7 markedly increased cellular migration and invasion in breast [14] and prostate [37] cancer cells. These results are consistent with our findings in chondrosarcoma cells. Clinical reports have also indicated that a high-expression level of BMP-7 may serve as a biomarker for metastasis and poor prognosis in various malignancies, such as esophageal cancer [38], lung cancer [18], gastric cancer [3], colorectal cancer [15], liver cancer [39], and melanoma [5].

BMPs belong to the TGF-β superfamily, which induces the signals through type I and type II BMP receptors. A previous study showed that the TGF-β and BMP signaling pathways were active in conventional central chondrosarcoma and those the activities were positively correlated to the histopathological grade [9]. Recently, targeting the TGF-β pathway holds promise as a novel therapeutic approach to prevent cancer metastasis [40], [41], [42]. Similarly, more recent research suggests that treatment with BMP receptor antagonists results in a reduction of cell migration and invasion, which may offer a promising novel strategy for cancer therapy, particularly metastasis [43], [44].

Previous reports have indicated that TGF-β and BMP-2, both highly homologous to BMP-7, are able to enhance cell motility and αv*β*3 integrin expression in human chondrosarcoma cells, via pathways involving PI3K, Akt, and NF-κB [23], [24]. Nevertheless, deregulation of integrin expression and/or signaling has been identified in many chondrosarcomas. Therefore, inhibition of integrin expression and signaling has been considered a promising approach in chondrosarcoma therapy because they are exposed on the cell surface and are sensitive to pharmacological blockade [1], [45].

In conclusion, we have explored the signaling mechanisms of BMP-7 in the regulation of αvβ3 integrin expression in human chondrosarcoma cells. Our results demonstrated that BMP-7 increases the expression of αvβ3 integrin by activating c-Src/PI3K/Akt/IKK/NF-κB signaling pathway, which may in turn enhance the binding of NF-κB transcription factor to the promoter of αvβ3 integrin, leading to the transactivation of αvβ3 integrin expression. These findings may provide a better understanding of the mechanisms underlying BMP-7 pathogenesis and can utilize this knowledge translationally for novel treatment strategies for chondrosarcoma.

## Supporting Information

Figure S1 **BMP-7 induced cell migration and integrin expression through c-Src/PI3K/Akt/NF-κB pathway in SW1353 chondrosarcoma cells.** Cells were pretreated with αvβ3 monoclonal antibody, PP2, Ly294002, wortmannin, Akt inhibitor, or PDTC followed by stimulation with BMP-7 for 24 h, and *in vitro* migration and αv or β3 integrin expression was measured by Transwell (A&B) and flow cytometry (C&D). Results are expressed as the mean ± SEM. *p<0.05, compared to basal expression levels. #*p*<0.05, compared to expression levels in the BMP-7-treated group.
(TIF)
