# Supplementary figures and images for: BMP-7 Enhances Cell Migration and αvβ3 Integrin Expression via a c-Src-Dependent Pathway in Human Chondrosarcoma Cells
Source: PLoS One. 2014 Nov 12;9(11):e112636. doi: 10.1371/journal.pone.0112636 (PMC4229252; doi:10.1371/journal.pone.0112636)

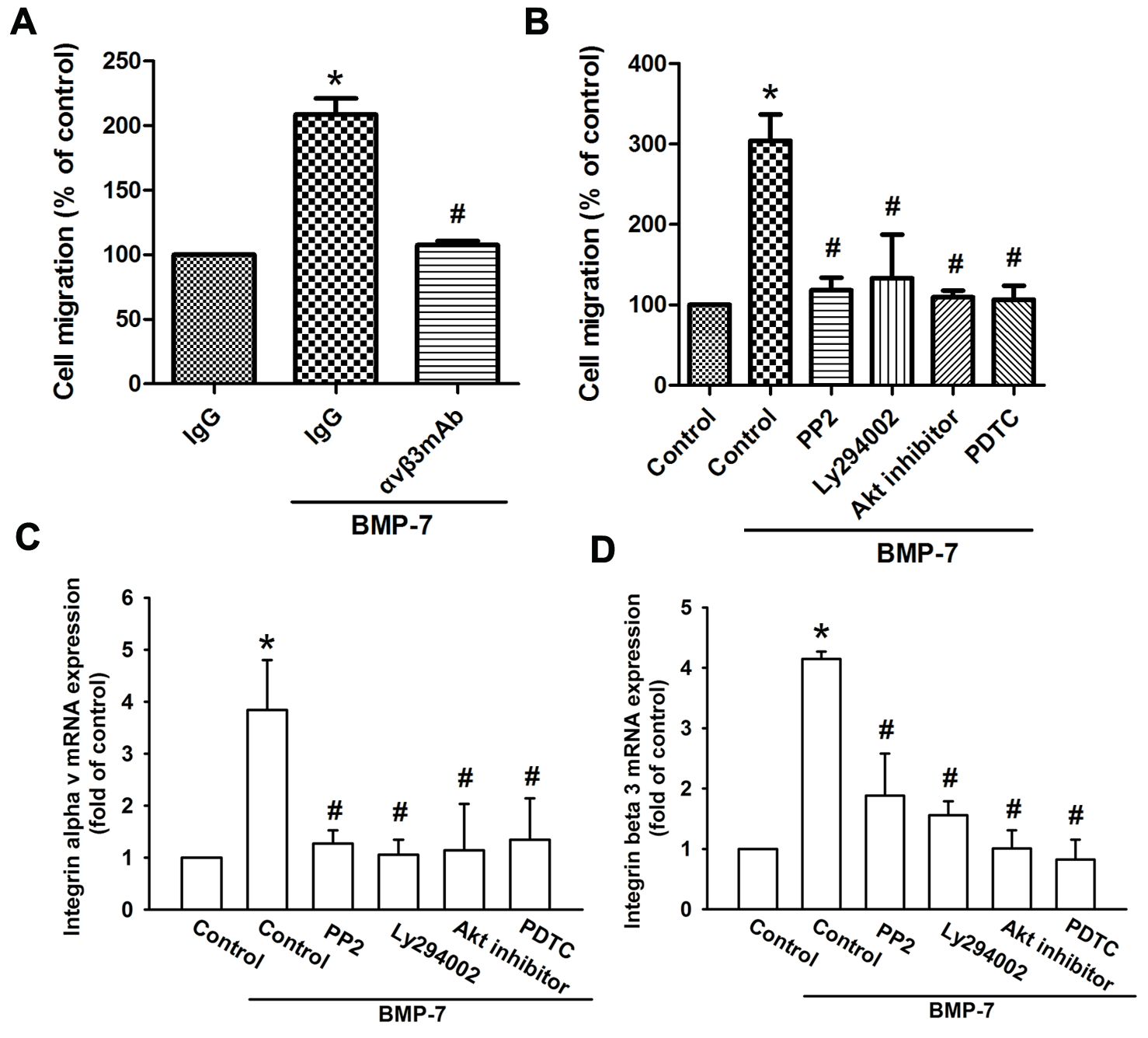

Supplement: Figure S1 — BMP-7 induced cell migration and integrin expression through c-Src/PI3K/Akt/NF-κB pathway in SW1353 chondrosarcoma cells. Cells were pretreated with αvβ3 monoclonal antibody, PP2, Ly294002, wortmannin, Akt inhibitor, or PDTC followed by stimulation with BMP-7 for 24 h, and in vitro migration and αv or β3 integrin expression was measured by Transwell (A&B) and flow cytometry (C&D). Results are expressed as the mean ± SEM. *p<0.05, compared to basal expression levels. #p<0.05, compared to expression levels in the BMP-7-treated group. (TIF) [file pone.0112636.s001.tif]
